# Deep learning-based motion artifact removal in functional near-infrared spectroscopy

**DOI:** 10.1117/1.NPh.9.4.041406

**Published:** 2022-04-23

**Authors:** Yuanyuan Gao, Hanqing Chao, Lora Cavuoto, Pingkun Yan, Uwe Kruger, Jack E. Norfleet, Basiel A. Makled, Steven Schwaitzberg, Suvranu De, Xavier Intes

**Affiliations:** a Rensselaer Polytechnic Institute, Center for Modeling, Simulation and Imaging in Medicine, Troy, New York, United States; b Rensselaer Polytechnic Institute, Department of Biomedical Engineering, Troy, New York, United States; c University at Buffalo, Department of Industrial and Systems Engineering, Buffalo, New York, United States; d U.S. Army Combat Capabilities Development Command–Soldier Center, Orlando, Florida, United States; e SFC Paul Ray Smith Simulation and Training Technology Center, Orlando, Florida, United States; f Medical Simulation Research Branch, Orlando, Florida, United States; g University at Buffalo, Department of Surgery, Buffalo, New York, United States

**Keywords:** functional near-infrared spectroscopy, motion artifact, deep learning, denoising autoencoder

## Abstract

**Significance:**

Functional near-infrared spectroscopy (fNIRS), a well-established neuroimaging technique, enables monitoring cortical activation while subjects are unconstrained. However, motion artifact is a common type of noise that can hamper the interpretation of fNIRS data. Current methods that have been proposed to mitigate motion artifacts in fNIRS data are still dependent on expert-based knowledge and the post hoc tuning of parameters.

**Aim:**

Here, we report a deep learning method that aims at motion artifact removal from fNIRS data while being assumption free. To the best of our knowledge, this is the first investigation to report on the use of a denoising autoencoder (DAE) architecture for motion artifact removal.

**Approach:**

To facilitate the training of this deep learning architecture, we (i) designed a specific loss function and (ii) generated data to mimic the properties of recorded fNIRS sequences.

**Results:**

The DAE model outperformed conventional methods in lowering residual motion artifacts, decreasing mean squared error, and increasing computational efficiency.

**Conclusion:**

Overall, this work demonstrates the potential of deep learning models for accurate and fast motion artifact removal in fNIRS data.

## 1 Introduction

Functional near-infrared spectroscopy (fNIRS) is a noninvasive neuroimaging technique to monitor brain activity indirectly. It measures the intensity of near-infrared light diffusely scattered through cortical tissues to quantify changes in oxyhemoglobin concentration (HbO) and deoxyhemoglobin concentration (HbR) with respect to cortical regions.^1^^–^^4^ HbO and HbR time series can reflect changes in neurovascular coupling and hence, neuronal activity. fNIRS has been widely used in cognitive,^5^^–^^9^ motor skill studies,^10^^–^^15^ and brain–computer interface technique.^16^

Although the fNIRS technology has been improved over the years, the processing of fNIRS data set can still be a challenging task. Especially, it is still difficult and time-consuming to identify and correct for motion artifacts caused by the changes in the coupling between optodes and scalp. Such artifacts are expressed as peaks or shifts in the time-series signals. Since the magnitudes of the peaks or shifts are usually much higher than the hemodynamic response function (HRF), the fNIRS signals are significantly contaminated and do not reflect the cortical activities. The phenomenon is more noticeable when the motion of the head and limbs are inevitable or even required in the experiment protocols, such as speeches,^17^ walking,^18^ and surgical tasks.^11^^,^^12^ The problem has been exacerbated by the recent rise of wearable or wireless fNIRS devices,^19^^,^^20^ which extend mobile ranges of these devices for tasks such as running or team working, that are more susceptible to motion artifacts. Thus, an efficient methodology to remove motion artifacts is essential to utilize fNIRS in those scenarios.

A few strategies that have been developed over the years include keeping any trials with motion artifacts during the data processing. This may be used only when large datasets are collected and is not the current predominant practice. Another strategy is to identify trials/channels with motion artifacts by visual inspection or to use functions such as *hmrMotionArtifact* function in the prevalent fNIRS data processing toolbox HomER2 and then discard them from further analysis. Though, the most appropriate methodology is to process these trials/channels using advanced time-series data processing methods. These include spline interpolation,^21^ wavelet filtering,^22^ principal component analysis (PCA),^23^ Kalman filtering,^24^ and correlation-based signal improvement (Cbsi).^25^ The performance of these methods, however, largely depends on a set of assumptions to describe motion artifacts and the subjective selection of associated tuning of parameters (Table 1). As an example, Ref. 29 demonstrated that selecting the PCA parameter, i.e., the percentage of variance in the data that PCA removed^27^ to be 0.80 and 0.97 produced significantly different results. Thus, a method that does not require the subjective fine-tuning of the parameters, or does not rely on stringent assumptions, is highly desirable. Here, we propose a deep learning method to learn the noise features automatically.

**Table 1:** The motion artifact removal models for fNIRS.

Model name and references	Assumptions	Implementation steps	Drawbacks
Spline interpolation^21^	The shape of the motion artifact is captured by spline interpolation.	1. Identify the noise.	Denoise performance depends on the noise detection method. The interpolation degree needs to be tuned.
		2. Model the noise by cubic spline interpolation.	
		3. Subtract the interpolation from the original signal.	
		4. Reconstruction.	
Wavelet filtering^22^^,^^26^	The wavelet coefficients are assumed to be normally distributed, and the outliers are accounted as motion artifacts.	1. Wavelet discrete decomposition.	Probability threshold alpha needs to be tuned.
		2. Identify the outliers in the coefficients larger than the probability threshold alpha.	
		3. Set the outliers to zeros.	
PCA^23^^,^^27^	The first several components of the PCA represent the variance caused by motion artifacts. Motion artifacts are likely to occur in most channels at the same time.	1. Apply PCA to produce uncorrelated components.	The number/portion of the components to be removed needs to be tuned. It is limited by the total number of channels available. As a spatial filtering method, PCA depends on the geometry of the probes.
		2. Remove the components that have the highest contribution to the variance of the original data.	
Kalman filtering^24^	The state is assumed to be motion-free.	1. Predict the state of the next time step.	Build-up errors may happen as prediction time increases.^28^
		2. Correct the prediction based on the measured signal.	
		3. Repeat steps 1 and 2.	
Cbsi^25^	HbO and HbR are negatively correlated. Motion artifacts are independent of Hb. The ratio between HbO and HbR is the same, irrespective of the presence of artifacts.	1. $\mathrm{Hb}O^{\prime }=\frac{\mathrm{HbO}-\alpha \cdot \mathrm{HbR}}{2}$,	The impacts of motion artifacts may differ between HbO and HbR.
		2. $\mathrm{Hb}R^{\prime }=-(\frac{1}{\alpha }\cdot \mathrm{Hb}O^{\prime })$, where α=std(HbO)/std(HbR).	

Over the last decade, deep neural networks have emerged as a powerful tool to suppress noise in image datasets in a fast and efficient manner. Deep learning models have been shown to produce competitive denoising results while retain more texture details when compared to conventional methods.^30^^–^^33^ Deep learning networks also showed superior performance when applied to medical imaging problems. For example, denoising autoencoder (DAE) model could denoise mammograms [structural similarity index measure (SSIM) from 0.45 to 0.73] and dental x-ray data (SSIM from 0.62 to 0.86).^34^ A DAE model achieved 10% higher peak signal-to-noise ratio (PSNR) and SSIM than the conventional algorithm in chest radiograms.^35^ A recent study^36^ showed that a long short-term memory (LSTM) network increased the accuracy of voxels classification in fMRI data by more than 20%. Another study^37^ showed that the deep learning model could completely remove the Gibbs phenomena in diffusion MRI data. DAE model has also been applied to denoising EEG data and increased PSNR values in EEG channels.^38^ A previous study^39^ employed artificial neural network model to optimize the weights of wavelet basis to denoise fNIRS data and achieved higher contrast-to-noise ratio (CNR) than conventional wavelet denoising methods. Another study^40^ detected motion artifact types using machine learning models on broadband fNIRS data.

Herein, we report on the use of a DAE model associated with a dedicated loss function purposely designed to remove the motion artifacts. To train such a deep learning network, we implemented an autoregression (AR) model to generate a large synthetic fNIRS dataset. The performance of our DAE methodology was established using this synthetic data set and benchmarked against the current conventional methods used by the fNIRS community. Moreover, the performances of the DAE were successfully validated on an open-access experimental dataset.

## 2 Methods

### 2.1 Data Collection for the Experimental Dataset

The data used in this study were adopted from a public available dataset.^41^ This fNIRS dataset contains motion artifacts (reading aloud, nodding their head up and down, nodding sideways, twisting right, twisting left, shaking head rapidly from side to side and raising their eyebrows) under resting state of eight participants. The details of this dataset can be found in Ref. 42. We also used a finger tapping dataset,^43^ which had not been seen by the model before to further test the ability of the model on a real task dataset.

### 2.2 fNIRS Data Simulation

**Fig. 1:**
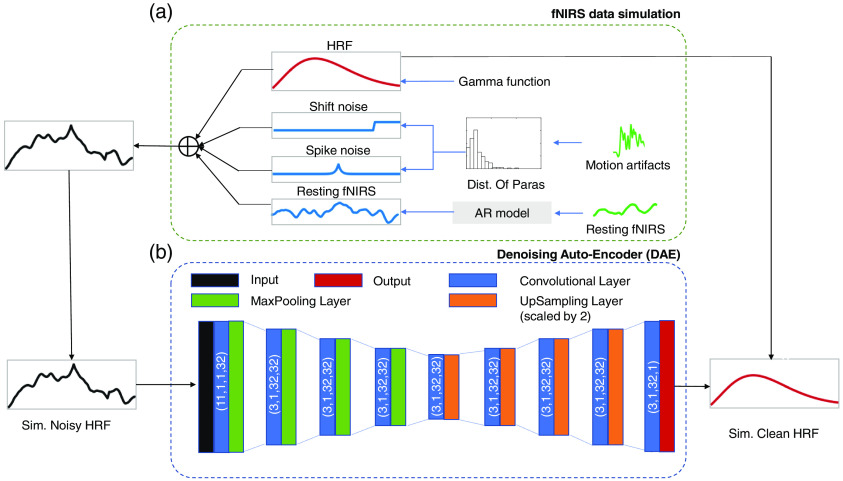
Illustration of the fNIRS data simulation process and the designed DAE model. (a) The green lines are the experimental fNIRS data, including noisy HRF and resting fNIRS data, while the blue and red lines are simulated ones. The AR models are fitted to the experimental resting-state fNIRS time series data, based on whose parameters the simulated resting fNIRS data are generated. The HRFs are simulated from gamma functions. The shift and spike noise are simulated based on the same distribution of the parameters from the experimental HRF. The simulated noisy HRF data (black line) is the sum of the simulated HRF, the shift noise, the spike noise, and the resting-state fNIRS. (b) DAE model: the input data of the DAE model are the simulated noisy HRF, and the output is the corresponding clean HRF without noise. The DAE model incorporates nine convolutional layers, followed by max-pooling layers in the first four layers and upsampling layers in the next four layers, with one convolutional layer before the output. The parameters are labeled in parentheses for each convolutional layer, in the order of kernel size, stride, input channel size, and kernel number.

The fNIRS data simulation procedure is presented in Fig. 1(a). The simulated noisy HRF data (F′(t)) consist of the superposition of three components: the clean HRF (F(t)), the motion artifact ($\Phi_{\mathrm{MA}}(t)$) and the resting-state fNIRS ($\Phi_{\mathrm{rs}}(t)$) components:

$$\underset{\mathrm{noisy}\;\mathrm{HRF}}{\underset{⏟}{F^{\prime }(t)}}=\underset{\mathrm{clean}\;\mathrm{HRF}}{\underset{⏟}{F(t)}}+\underset{\mathrm{motion}\;\mathrm{artifacts}}{\underset{⏟}{\Phi_{\mathrm{MA}}(t)}}+\underset{\mathrm{resting}-\mathrm{state}\;\mathrm{fNIRS}}{\underset{⏟}{\Phi_{\mathrm{rs}}(t)}}.$$ (1)

We simulated the clean HRF component (F(t)) by gamma functions as suggested in steps in Ref. 44. Instead of using 54  μM·mm in all the simulated HRF, we were randomly selecting values from a uniform distribution between 30 and 80  μM·mm, such that the DAE could learn from a variety of learning samples. The HbR amplitude is always half of the HbO amplitude. The motion artifacts ($\Phi_{\mathrm{MA}}(t)$) consisted of two types: spike noise and shift noise. Spike artifacts were simulated as Laplace distribution function^45^ given as

$$f(t)=A\cdot \exp(-\frac{\left|t-t_{0}\right|}{b}),$$ (2)

where A represents the peak amplitude, b represents the scale parameter, and $t_{0}$ represents the time point of the “peak.” Shift noise was simulated as a positive or negative change in DC value. All the parameters to determine noise functions were first derived from the experimental fNIRS dataset. The parameters for each artifact were then drawn from the parameter values in the experimental data. As described in Ref. 45, the resting-state fNIRS ($\Phi_{\mathrm{rs}}(t)$) was simulated using an AR model that included five lagged terms. We first fitted an AR model to the experimental resting-state data to determine the model parameters. Then, these parameters were used to simulate the resting-state fNIRS.

### 2.3 Denoising Autoencoder Model Design

The DAE concept was proposed by Vincent et al.,^46^ and it has numerous applications. For our purpose, two essential design criteria needed to be established: architectural hyperparameters and specific loss function. Based on prior knowledge and empirical evidence, our final DAE model consisted of the nine stacked convolutional layers shown in Fig. 1. The convolutional layers were followed by max-pooling layers (the first four layers), followed by the next four upsampling layers, and one more convolutional layer before the output. A smaller network topology containing only four layers produced a significant reduction in performance (see our previous work^47^). The network was trained using backpropagation to minimize the loss function. The time-series data of the simulated noisy HRF (F′(t)) was the input data, and the output data were the simulated clean HRF (F(t)).

The loss function was specially designed for this problem. First, the loss function minimized the discrepancy between the predicted fNIRS data with the ground truth data. For this purpose, we adopted the mean squared error (MSE) loss function ($L_{\mathrm{mse}}$) here:

$$L_{\mathrm{mse}}=\frac{1}{n}\sum_{}^{}({\hat{y}}_{i}-y_{i}{)}^{2},$$ (3)

where $y_{i}$ represents ground-truth value and ${\hat{y}}_{i}$ represents the predicted value.

Next, the loss function minimized the total variation of the predicted signal:

Lvar=1n∑(y^i−u^)2, (4)

where $\hat{u}$ is the mean value of ${\hat{y}}_{i}$.

Finally, the loss function minimized the number of motion artifacts in the output data, which we evaluated using the *hmrMotionArtifact* function in HomER2. The motion artifact detection is twofold: (i) the standard deviation (std) exceeds the standard deviation threshold or (ii) the amplitude change (amp) exceeds the amplitude threshold. Here, we designed the standard deviation loss function ($L_{\mathrm{std}}$) for (i) and the amplitude loss function ($L_{\mathrm{amp}}$) for (ii), as described in Eqs. (5)–(8):

$$L_{\mathrm{std}}=\frac{1}{N_{\text{std}}}\sum_{i=1}^{T}\sum_{j\in j|\Delta_{i}dc_{j}>m}\Delta_{i}dc_{j},$$ (5)

$$L_{\mathrm{amp}}=\frac{1}{N_{\mathrm{amp}}}\sum_{i=1}^{T}\sum_{j\in j|\Delta_{i}dc_{j}>C_{\mathrm{amp}}}\Delta_{i}dc_{j},$$ (6)

where

$$m=\sigma_{\Delta_{1}\mathrm{dc}}\cdot C_{\mathrm{std}},$$ (7)

$$\Delta_{i}dc_{j}=dc_{j}-dc(j+1).$$ (8)

In the above equations, dc is the projected optical intensity value derived from the predicted hemoglobin value ${\hat{y}}_{i}$ via the Beer–Lambert Law dci=(y^i/εd), where ε is the attenuation coefficient, and d is the photo path length. $N_{\mathrm{std}}$ represents the number of all the time points in 1∼T and $\Delta_{i}dc_{j}>m$; while $N_{\mathrm{amp}}$ represents the number of all the time points in 1∼T and $\Delta_{i}dc_{j}>C_{\mathrm{amp}}$. $\sigma_{\Delta_{1}}dc$ is the standard deviation of changes in dc in one time step. $C_{\mathrm{amp}}$ and $C_{\mathrm{std}}$ were motion detection thresholds in amplitude and standard deviation changes. Those values work as the same role as ${\mathrm{AMP}}_{\mathrm{thresh}}$ and ${\mathrm{STDEV}}_{\mathrm{thresh}}$ in the HomER2 function *hmrMotionArtifact*. If the signal changes by more than $C_{\mathrm{amp}}$ over the time interval, then this time point is marked as a motion artifact. If the signal changes by more than ${\mathrm{STDEV}}_{\mathrm{thresh}}*\mathrm{stdev}(d)$ over the time interval, then this time point is marked as a motion artifact. The goal of $L_{\mathrm{std}}$ and $L_{\mathrm{amp}}$ is to decrease the amplitudes of the “jump/peak” signal that are defined as motion artifacts by the threshold values of $C_{\mathrm{amp}}$ and $C_{\mathrm{std}}$. The users can change those values to change their criteria to define and detect motion artifacts as they would do in homer2.

Finally, the loss function to train the deep learning network (Loss) is a linear combination of the four individual loss functions above [Eqs. (3)–(6)] with hyperparameters, $\theta_{1}$, $\theta_{2}$, $\theta_{3}$ as shown as

$$\mathrm{Loss}=L_{\mathrm{mse}}+\theta_{1}\times L_{\mathrm{var}}+\theta_{2}\times L_{\mathrm{std}}+\theta_{3}\times L_{\mathrm{amp}},$$ (9)

where $\theta_{1}$, $\theta_{2}$, $\theta_{3}$ were set to be 1, 1, and 10, respectively, to balance the magnitude of each term.

### 2.4 Model Training

We trained our DAE model using the PyTorch package in Python 3. We adopted a leave-one-subject-out cross validation scheme by simulating data from experimental data from all subjects but one and then tested the model on the one subject that has been left out. We repeated this procedure until we tested all the subjects. We randomly split the simulated dataset into training, validation, and testing datasets in the ratio of 8:1:1. The model was trained on the training dataset and validated on the validation dataset. After completing the model training, its performance was evaluated using the testing dataset. The learning rate was set at 0.0001 and divided by 10 every 25 epochs. The model was trained for 100 epochs, and the parameters were saved at the epoch corresponding to the least validation loss. The optimization method was “ADAM.”^48^ After successful training and validation on the synthetic datasets, the DAE model was applied to the experimental dataset.

### 2.5 Conventional Denoising Methods

We compared the performance of our deep learning denoising network to models that we obtained by applying the following competitive methods that have recently been proposed in the literature: (i) spline interpolation, (ii) wavelet filtering, (iii) PCA, (iv) Kalman filtering, and (v) Cbsi. These methods have been widely used in the fNIRS community, and we implemented these functions using HomER2.^49^ Each method, summarized in Table 1, has been thoroughly discussed, analyzed, and compared in two review papers.^29^^,^^44^ Table 1 also summarizes the assumptions imposed on each method and briefly discusses their known drawbacks. The data flow of the data processing of these conventional denoising methods and DAE is presented in Fig. S1 in the Supplemental Material. All methods are performed before block average. The motion artifacts were detected prior to spline by *hmrMotionArtifactByChannel* function from homer2. We detected the motion artifacts in the OD signals by *hmrMotionArtifact* for all the methods. For CBSI and DAE, since they work on concentration values, we first converted it to OD value by *hmrConc2OD* in homer2 and use *hmrMotionArtifact* to detect motion artifacts. The standard deviation threshold (${\mathrm{STDEV}}_{\mathrm{thresh}}$ in homer2) is set to 20 and the amplitude threshold (${\mathrm{AMP}}_{\mathrm{thresh}}$ in homer2) is set to 0.3 to detect the motion artifacts in this particular work. But users can change these values according to suggestions in homer2 or their experience. For PCA, we were using target recursive PCA.^27^ After the sensitivity analysis, we ended up selecting 0.97 for PCA threshold values and 0.75 for wavelet method (see more details in Sec. 3.2). For Kalman filters, the initial state vector $x_{0}$ were set as the first values of the signals, and the initial error covariance matrix $P_{0}$ is set as the square of the first values of the signals.

### 2.6 Metrics for Model Evaluation

To evaluate the denoising performance for each model when applied to the simulated and the experimental dataset, we used the number of motion artifacts remaining after applying the models measured by the *hmrMotionArtifact* function in HomER2. The fewer the motion artifacts remaining, the better the model performance. The number of motion artifacts could not reflect how well the methods could reserve the HRF in it, so we introduce another metrics as MSE to access the ability to reserve the true HRF of each model. To further assess the processing speed, we also employed computation time. The experimental data we adopted^42^ are the resting state data with various purposefully added motion artifacts. There is no brain activation in this dataset. We added stim markers every 73.142 s and then we labeled this dataset as “No act.” in this paper. So in this “No act.” dataset, the HRF should be zeros because there is no brain activation involved. Then we added synthetic HRF (modified gamma function of amplitude of 54  μM·mm), to the stim marker locations and labeled this dataset as “Act.” dataset. In this “Act.” dataset, the ground truth HRF should be a modified gamma function with amplitude of 54  μM·mm. We used the software G*Power to do power analysis to control type-II error. The power value of the testing dataset is 1.00; the power of No act. experimental dataset is 1.00; the power of Act. experimental dataset is 0.99.

### 2.7 Sensitivity Analysis

To have a fair comparison, we determined the parameters for the competitive methods to establish models that achieve the best performance. In practice, the parameters could be adjusted by a visual check of each trial data. However, for the large dataset we had in this study, a more automatic method was required. Here, we adopted the sensitivity analysis method^44^ to identify the best set of parameters. For spline interpolation, the interpolation parameter ($p_{\mathrm{Spline}}$) was varied from 0 to 1; for wavelet filtering, the probability threshold ($iqr_{\mathrm{Wav}}$) was varied from 0.1 to 1.5.^50^ The evaluation metrics for these models were MSE and the number of motion artifacts left after applying the models.

## 3 Results

### 3.1 Data Simulation

**Fig. 2:**
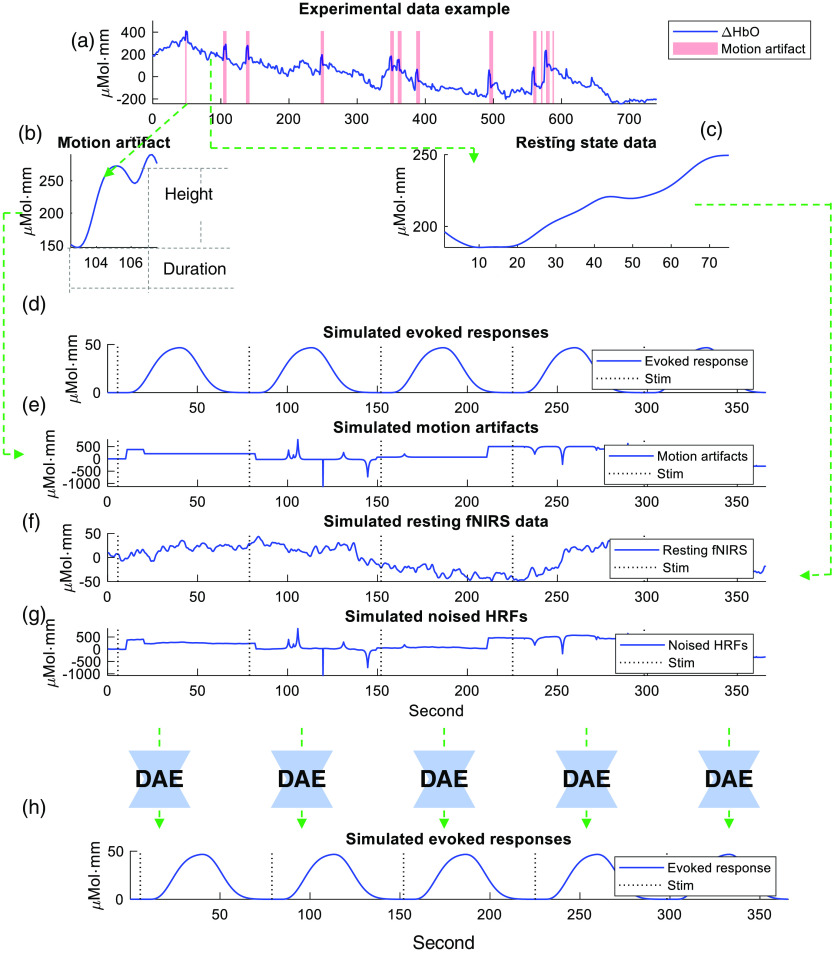
An example of the fNIRS data simulation process and the designed DAE model. (a) An example of experimental data. (b) The model artifact extracted from the experimental data in (a). (c) The resting state period extracted from the experimental data in (a). (d) Simulated evoked responses. (e) Motion artifact data simulated based on the parameters extracted from the motion artifact in (b). (f) Resting state data simulated based on the data in (c). (g) Synthetic noised HRFs, which is the sum of data in (d)–(f). (h) The expected output of DAE model, which is the same with the data in (d).

In the dataset we used to train, there were seven subjects, with one run for each subject. We generated 2000 runs of resting data from each subject’s experimental data. We added five trials of HRFs onto each run and added no HRFs to 200 runs out of the 2000 runs to form the training dataset. In other words, we generated 2200 runs from each subject and each run had five trials. Since our model denoised channel by channel, the number of channels was one here. For each round of training of the leave-one-subject-out crossover scheme, we left one subject’s data out. Then, we had 77,000 training trials in total. Figure 2 shows an example of how we extracted motion artifact period and resting period from experimental data, how we determined the shape of the motion artifacts (e.g., height and duration) then simulated motion artifacts based on those parameters, and how we simulated resting fNIRS data based on resting state period data and then synthesize the simulated data. The DAE was applied on each trial before the trial average. The SNRs of the experimental data and the simulated data are presented in Fig. S3 in the Supplemental Material; the SNR values of simulated data are comparable to the experimental data.

### 3.2 Sensitivity Analysis

The results of the sensitivity analysis are shown in Fig. S2 in the Supplemental Material. For PCA, $\sigma_{\mathrm{PCA}}=0.99$ yielded the lowest MSE and number of motion artifacts. In the literature, $\sigma_{\mathrm{PCA}}=0.97$ was selected in Refs. 29 and 44. In this work, we kept 0.97 for $\sigma_{\mathrm{PCA}}$. For spline interpolation, $p_{\mathrm{Spline}}=0.99$ yielded the lowest MSE and number of motion artifacts and was also selected in Refs. 21, 29, and 44. Thus, $p_{\mathrm{Spline}}=0.99$ was selected in this work. For wavelet filtering, we selected $iqr_{\mathrm{Wav}}=0.75$ to keep both MSE and number of motion artifacts at a low level.

### 3.3 Comparison of Models on Denoising Performance

**Fig. 3:**
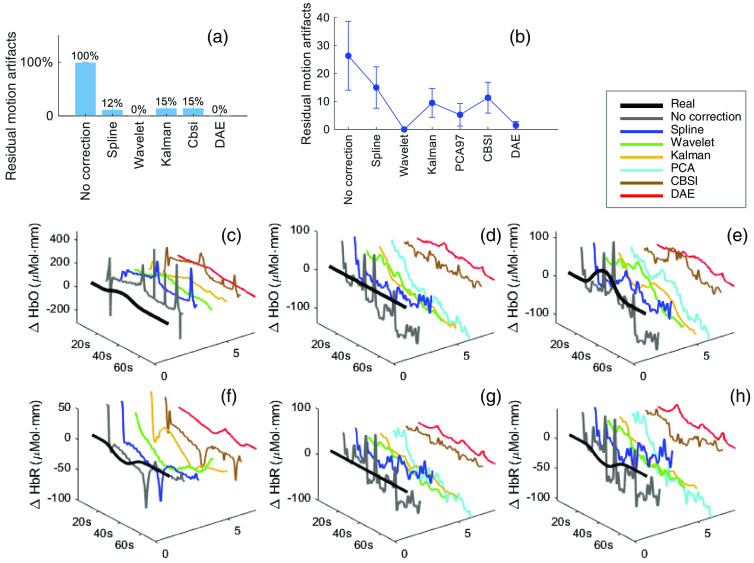
The denoising results. (a) The number of residual motion artifacts for the simulated testing dataset. (b) The number of residual motion artifacts for experimental data. (c), (f) An example of processed data by different models in the simulated dataset, (d), (g) in experimental data under “No act.” condition, and (e), (h) under “Act.” condition. “No correction” indicates that no motion artifact correction model was used. An enlarged two-dimensional (2D) view of (c)–(h) is in Fig. S4 in the Supplemental Material.

We applied each model except PCA on the simulated testing dataset and derived the number of residual motion artifacts. All the motion artifact removal models reduced the number of motion artifacts. Using our DAE model resulted in the minimum number of motion artifacts, with 100% of the motion artifacts removed [Fig. 3(a)]. We then applied each model on experimental data [Fig. 3(b)]. Here, on experimental data, the DAE model also derived the second minimum residual motion artifacts next to the wavelet.

**Fig. 4:**
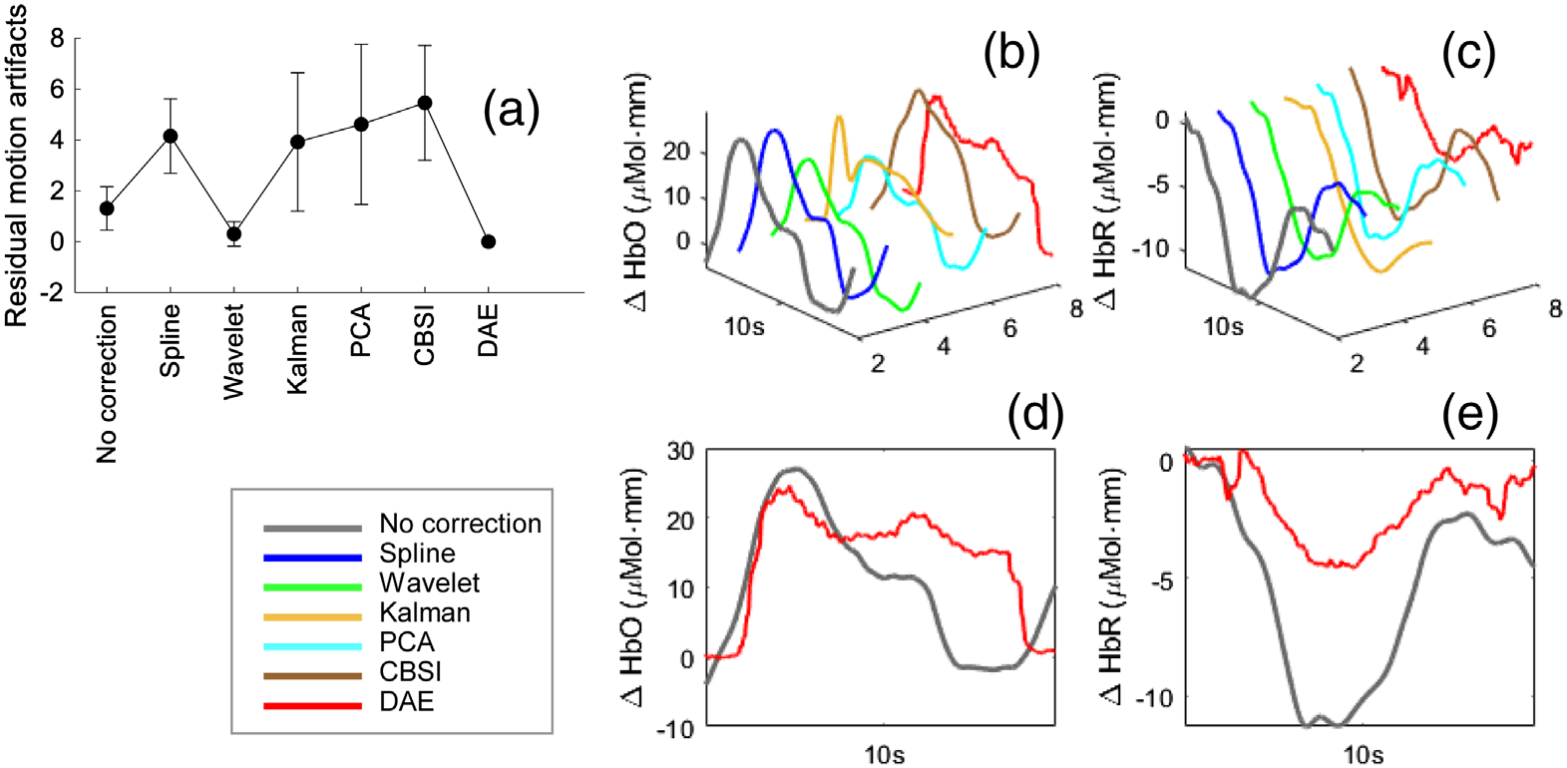
The denoising results in the new dataset. (a) The number of residual motion artifacts for experimental data. (b), (c) An example of processed data by different models. (d), (e) An enlarged 2D view of (b) and (c).

We further visualized examples from simulated and experimental data in Figs. 3(c)–3(h). From the examples, the DAE model smoothed the signal while keep the evoked feature of the HRFs. We further tested our model on a new, publicly available finger-tapping dataset that has never been seen by the model. The testing results are shown in Fig. 4. We can see the residual motion artifact of DAE model is lower than other models [Fig. 4(a)]. The recovered HRF shape in the motor region was also acceptable by visual check [Figs. 4(b)–4(e)].

The MSE values based on HbO for simulation testing dataset, experimental dataset are presented in Table 2 (the results based on HbR are in Table S1 in the Supplemental Material). The DAE model achieved the lowest MSE in all the datasets. The MSE values were statistically analyzed comparing between DAE model and each other model. We used paired t-test if the samples were normal distributed (tested by Kolmogorov–Smirnov test) and paired sign test if the samples were not normal distributed. The significance level was set at 0.05.

**Table 2:** The MSE based on HbO. The median value and the IQR value of MSE for each model and the p-values in the comparison between each model and DAE.

Median (IQR) ($(\mu \mathrm{Mol}\cdot \mathrm{mm}{)}^{2}$)	Sim. testing	Sig. test	Real testing (w/o act.)	Sig. test	Real testing (act.)	Sig. test
No correction	9086.23 (29798.72)	*p* = 0.000	10,663.50 (37,293.42)	*p* = 0.000	10,658.95 (37,320.75)	*p* = 0.000
Spline	3699.08 (9695.42)	*p* = 0.000	11,483.56 (37,471.50)	*p* = 0.000	11,238.91 (35,722.56)	*p* = 0.000
Wavelet	4023.11 (18,566.72)	*p* = 0.000	4438.63 (12524.89)	*p* = 0.000	4500.36 (13281.81)	*p* = 0.000
Kalman	7630.74 (31,175.33)	*p* = 0.000	6309.14 (22,436.39)	*p* = 0.000	6648.38 (23,806.81)	*p* = 0.000
PCA	—	—	11,362.13 (47,714.05)	*p* = 0.000	10,717.19 (48,408.62)	*p* = 0.000
Cbsi	4273.20 (15,380.82)	*p* = 0.000	3899.61 (22613.30)	*p* = 0.000	4198.70 (22,892.76)	*p* = 0.000
DAE	144.19(214.42)	—	226.81(121.91)	—	296.55(58.60)	—

The computation time was also recorded and is presented in Table 3 (CPU: Intel® Core™ i9-9900K CPU @ 3.60 FHz). The DAE model achieved the shortest computational times, with Cbsi as the second and PCA as the third. This result is expected as DAE, Cbsi, and PCA only perform matrix multiplication. The spline method is no more than cubic interpolation, but it requires a prior motion artifact detection step, which makes it slower than expected. Notably, wavelet methods are computationally costly.

**Table 3:** The computation time for each model on testing data.

Model	Computation time (s)
Spline	8.7
Wavelet	1202.1
Kalman	76.3
PCA	6.9
Cbsi	4.9
DAE	2.4

## 4 Discussion and Conclusion

We introduced a deep learning DAE network to suppress motion artifacts in recorded fNIRS data. To train a DAE network, we used simulated data from an AR model. We validated the efficiency of the trained DAE network by comparing its performance to models obtained from other conventional modeling methods when applied to both simulated and experimental fNIRS data. The data results showed that the DAE model yielded the lowest residual motion artifacts and MSE compared with competitive models. The DAE model was also the fastest in computation.

The limitation of labeled training data often jeopardizes the effectiveness of deep neural networks.^51^ Self-supervised learning^52^^,^^53^ has been widely used to overcome this limitation. Specifically, various pretext tasks have been used to train the deep network,^54^ such as colorizing grayscale images,^55^ image inpainting,^56^ and image jigsaw puzzle.^57^ In these tasks, inputs and labels were synthesized from the unlabeled training set. Then, these synthetic samples were used for training. In the work of Ref. 44, an fNIRS synthesis method was proposed as adding known HRFs to experimental resting-state data. However, this method was limited by the experimental resting-state fNIRS data available. As in our problem, we introduced a similar strategy to train the proposed deep network, but by simulating the resting-state fNIRS data by the AR model. Eighty thousand samples were simulated in this work to be able to cover the large range of HRF and noise parameters obtained from the experimental dataset. The trained DAE model was demonstrated to be efficient in fNIRS denoising. In the future, this fNIRS data synthesis scheme could be harnessed to develop more powerful deep learning models of fNIRS data processing. When applied to other real datasets, the range of HRF amplitude in the training data could be adjusted to the specific range. For example, in this paper, the range was predefined as 30 to 80  μM·mm and the ground truth (simulated HRF amplitude) in real data is 54  μM·mm. So the true HRF is covered by the predefined HRF range. However, in most cases when we do not know what the true HRF amplitude is, we suggest to use a larger predefined HRF range. Other than that, larger training sample size would also give optimized performance.

We simulated our dataset aiming to train our DAE model in this work. However, the synthetic dataset might be biased unfavorably to other models here by violating their assumptions. For example, PCA depends heavily on the covariance of motion artifacts across the channels. Our simulated dataset was not designed to have multiple motion artifacts across the channels at a similar time point. Thus, we did not apply PCA onto our simulation dataset.

We would also like to mention that we did not simulate our data in a way that the motion artifacts were locked with the evoked signal, which is a feature of motion artifacts in some study paradigms. Our simulated motion artifacts were randomly distributed along the time regardless of the stimulus. In the case that motion artifacts are locked with the stimulus, general linear model (GLM) could be a better option, since it is intrinsically robust to motion artifact locked by the evoked signal. Also, GLM is capable of including variety of regressers, such as short separator and accelerometer data to directly regress out motion artifacts. Even though we did not consider this specific scenario herein, it could be the focus of future work, including the use of DAE model incorporating short separator and accelerometer.

Multiple models have been suggested in the past to remove motion artifacts in fNIRS data. However, the effectiveness of each model depends on the underlying assumptions and the experimental protocol. For example, PCA has been used to remove systemic physiology and motion artifacts that were common across the channels in infant fNIRS data.^58^ Still, the effects changed with the extent of principal component filtering.^58^ In another study,^27^ targeted PCA performed better than wavelet filtering and spline interpolation when the motion artifacts were temporally independent of the stimuli. Since our data simulation method did not guarantee the covariance of motion artifacts across the channels, we did not expect PCA would work well on our simulated data. PCA is also sensitive to parameter changes. Cooper et al.^44^ compared PCA, wavelet, and spline on a synthetic HRF dataset. Their results demonstrated that spline produced the most significant improvement for MSE and $R^{2}$, while wavelet analysis produced the highest increase in CNR. Our simulation results also showed that the spline and wavelet models decreased MSE. In another study,^29^ wavelet and Cbsi were shown to be most effective in a real speech study where motion artifacts coincided with stimuli. However, Cbsi did not show superior performance here.

It is also worth mentioning that motion artifact correction is also possible using hardware design changes. For example, an acceleration sensor could be used in experiments to capture the motion data.^59^^,^^60^ Short separation detectors were proposed to remove the physiological artifacts^61^^–^^65^ and could also be used to remove motion artifacts.^66^ It could also be combined with other filter models to better remove motion artifacts.^67^ However, our work did not consider such hardware designs. Coupling of DAE with hardware solutions may be of interest in future studies.

Though real-time motion artifact removal was not the goal of this study, such requirements have been considered in Refs. 68 and 69. Our research indicates the superiority of DAE compared to other models with regard to computational speed. However, the design and software implementation of our current DAE model were not focusing on computational efficiency. It is well appraised that increased network complexity leads to increased inference time. Hence, future studies could focus on optimizing network architecture toward increased computational efficiency. Moreover, the custom GPU implementation of the DAE model is expected to greatly improve inference speed. Last, deep learning models, such as LSTM, could be used for real-time application.

In conclusion, we demonstrated that our DAE model has promising performances in lowering the number of motion artifacts and decreasing MSE metrics. To train the DAE model, we suggested an fNIRS synthesis method to generate a large amount of fNIRS data. The results showed the potential suitability and superiority of DAE for fNIRS motion artifact removal.

## Code Availability

The code associated with this manuscript is available at https://github.com/YuanyuanGao216/fNIRS_denoise_by_DL
