# Supplementary material for: Deep learning-based motion artifact removal in functional near-infrared spectroscopy
Source: Neurophotonics. 2022 Apr 23;9(4):041406. doi: 10.1117/1.NPh.9.4.041406 (PMC9034734; doi:10.1117/1.NPh.9.4.041406)
Supplement: Supplementary file 1 [file NPh_009_041406_SD001.pdf]

Supplementary information

Deep Learning-based Motion Artifact Removal in Functional Near-Infrared Spectroscopy (fNIRS)

Gao et al.

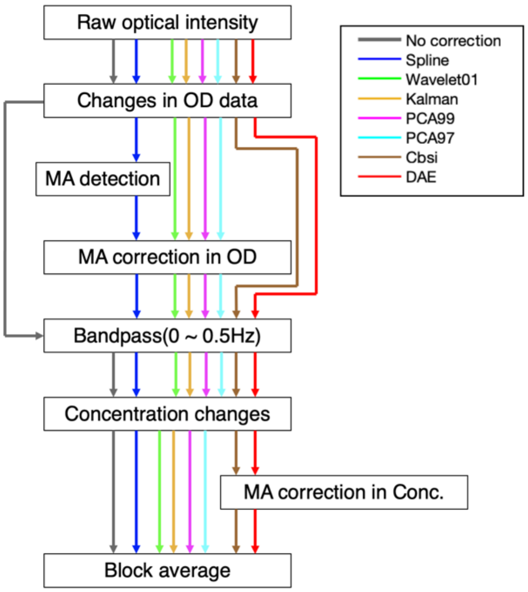

Fig. S1. Signal processing steps for all techniques.

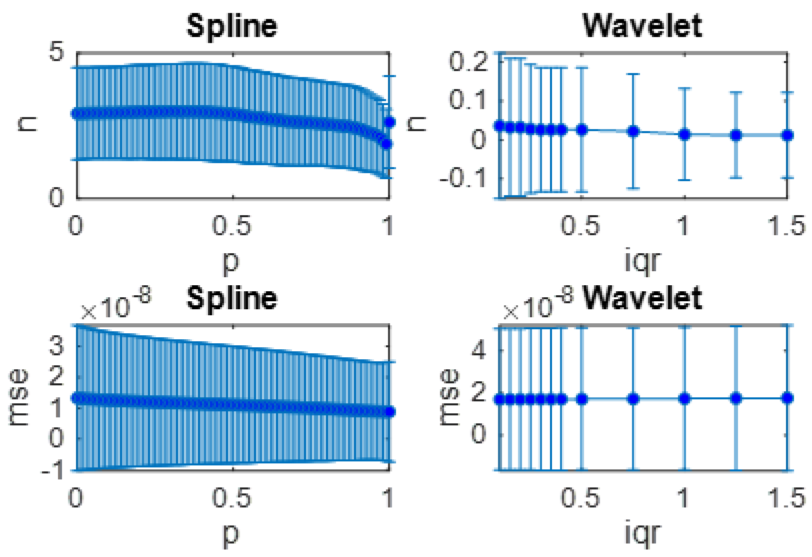

Fig. S2. Sensitivity analysis of parameters, p_{Spline}, $iqr_{wavelet}$ for spline interpolation and wavelet filtering methods respectively. MSE represents the mean squared error; N represents the number of motion artifact left after applying the models. The error range is the standard deviation.

Table S1. The MSE based on HbR. The median value and the IQR value of MSE for each model and the p-values in the comparison between each model and DAE.

Median (IQR) ($(\mu Mol \cdot mm)^2$)	Sim. testing	Sig. test	Real testing (No act.)	Sig. test	Real testing (Act.)	Sig. test
No correction	2943.90 (9542.44)	p = 0.000	2082.71 (11755.26)	p = 0.000	2082.77 (11756.03)	p = 0.000
Spline	1012.47 (2986.52)	p = 0.000	2325.50 (13591.18)	p = 0.000	2299.80 (13488.00)	p = 0.000
Wavelet	1128.03 (5754.46)	p = 0.000	736.74 (6739.76)	p = 0.000	739.20 (6716.76)	p = 0.000
Kalman	2286.17 (10032.05)	p = 0.000	1392.85 (11797.19)	p = 0.000	1475.68 (12008.45)	p = 0.000
PCA	-	-	2409.90 (10044.42)	p = 0.000	2361.82 (11919.84)	p = 0.000
Cbsi	1335.70 (4901.36)	p = 0.000	972.50 (5810.30)	p = 0.000	930.31 (5217.72)	p = 0.000
DAE	35.55 (72.51)	-	171.63 (186.60)	-	369.26 (279.27)	-

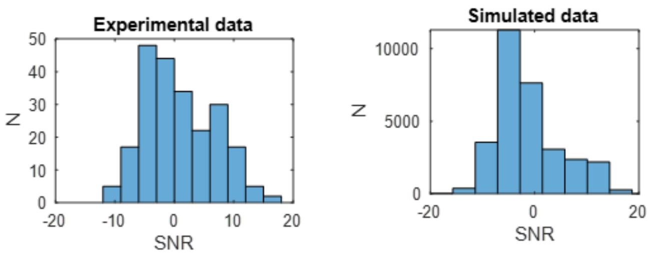

Fig. S3. The histograms of SNR of experimental data and simulated data.

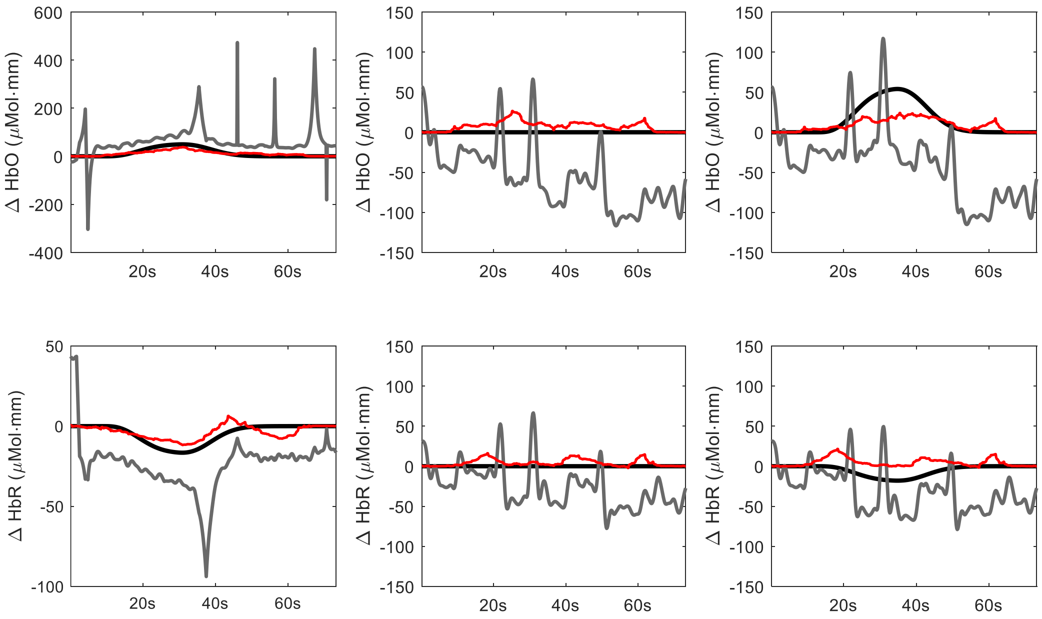

Fig. S4. The enlarged 2D view plot for Fig. 3(C-H) accordingly.
